# INCA-M: Mexican Adaptation of the Inventory of Callous-Unemotional Traits and Antisocial Behavior

**DOI:** 10.3389/fpsyg.2020.00753

**Published:** 2020-04-21

**Authors:** Fabia Morales-Vives, Mariana Gómez-Herrera, Andreu Vigil-Colet

**Affiliations:** ^1^Department of Psychology, Research Center for Behavior Assessment, Universitat Rovira i Virgili, Tarragona, Spain; ^2^Department of Psychology, National Autonomous University of Mexico, Mexico City (Ciudad de México), Mexico

**Keywords:** psychopathy, callous-unemotional traits, adolescents, social desirability, acquiescence, adaptation

## Abstract

Callous-unemotional traits are considered to be precursors of psychopathy, and are related to behaviors such as aggression, delinquency, antisocial behavior, and bullying in adolescents. For this reason, it is important to study these traits in childhood and adolescence with appropriate and reliable instruments. The aim of the current study is to develop a Mexican adaptation of the Inventory of Callous-Unemotional Traits and Antisocial Behavior (INCA) because few questionnaires in Spanish assess these traits, and even fewer have been validated for the Mexican population. The INCA questionnaire, developed in Spain, assesses the same three factors as the ICU questionnaire (unemotional, callousness, and uncaring), and it includes an additional factor of antisocial behavior with items on challenging authority and breaking social rules. It controls two response biases: social desirability and acquiescence. We administered the Mexican adaptation, named INCA-M, to 699 adolescents aged between 12 and 18 years old. Factor analysis yielded three dimensions, because most of the items referring to uncaring and antisocial behavior loaded on a common factor, which can be explained by cultural differences. We decided to remove these items of antisocial behavior so as to maintain the same three factors assessed by the ICU questionnaire. The results suggest that the INCA-M has good psychometric properties, with high factor simplicity and good reliability. Moreover, we found the expected correlations with impulsivity and the Big Five subscales, and also with the equivalent subscales assessed by the ICU questionnaire.

## Introduction

Psychopathy is a personality construct characterized by the manipulation of others for personal gain, a lack of empathy, and a lack of guilt and remorse (Viding et al., 2014; Duran-Bonavila et al., 2017). The disorder has had a great impact on society, because of its relationship with violent and antisocial behavior, and the connotations the word has acquired through movies and mass media. Although there is a debate about some of the issues related to this construct, in general authors do agree on its most important characteristics (Hare et al., 2000). In fact, according to Hare (2003), these features can be classified into four different areas: (a) An interpersonal area that involves being insensitive, arrogant, domineering and manipulative; (b) An affective area that involves a lack of empathy or regret, which affects the development of strong emotional bonds; (c) An impulsive lifestyle characterized by irresponsibility and stimulation seeking, and (d) Antisocial features that involve ignoring and violating social norms, juvenile delinquency and early behavior problems.

Callousness-unemotional (CU) traits are considered to be precursors of psychopathy, they can be observed at early ages (Viding et al., 2014), and remain relatively stable throughout life (Lynam, 1996; Frick et al., 2003b). They involve features such as the lack of empathy, manipulation of others, lack of remorse, irresponsible attitudes, and poor emotional expression (Frick, 2004). CU traits are related to many dysfunctional behaviors in children and adolescents, such as antisocial behavior and aggression (Frick et al., 2003a, 2005), substance-abuse related delinquency (Taylor and Lang, 2006), bullying (Thornberg and Jungert, 2017), etc. Some studies suggest that the antisocial behaviors displayed by adolescents with CU traits may be linked to deficits in reactivity to unpleasant emotional stimuli such as anxiety or fear (Blair et al., 2001; Kimonis et al., 2006; Frick and Viding, 2009). In fact, it seems that they are less inhibited by anxiety and fear (Frick et al., 1999, 2003b; Lynam et al., 2005) and are less sensitive to punishment (Fanti et al., 2016). More specifically, according to Pardini (2006), callousness mediates the relationship between violence and low fearfulness. In other words, low levels of anxiety and fear of punishment are related to serious violent delinquency, but high levels of callousness mediate the relationships. For this reason, it is important to study these CU traits in childhood and adolescence with appropriate and reliable instruments so that interventions targeting minors with these traits can be developed.

The questionnaire most commonly used for assessing CU traits is the Inventory of Callous-Unemotional traits (ICU), developed by Frick (2004) who regarded it as a unidimensional test comprised of items related to Careless, Unemotional, Uncaring, and Callousness. Nevertheless, he did not assess its unidimensionality. Various authors have studied the factor structure of the ICU, but the results are far from clear and show various problems.

Inventory of Callous-Unemotional traits factor structure was first studied by Essau et al. (2006). They performed an exploratory factor analysis and used the scree-test to propose a three-factor structure comprised of the factors Callousness, Uncaring, and Unemotional. They tried to confirm this factor structure, but they failed to get an acceptable fit until they introduced a model with two factors that enabled all the items to load onto a general Callous-Unemotional factor in addition to their loads on their corresponding scale. Furthermore, they only reached this fit after allowing 25 error terms to correlate on the basis of modification indices, which gives rise to serious doubts about the generalizability of the model to other samples. Another problem is the content of the scales, because different scales contain items with a very similar content (Morales-Vives et al., 2019).

Several studies have tried to confirm this structure but they either failed or had to make modifications such as removing certain items (Kimonis et al., 2008; López-Romero et al., 2015), removing some factors (Feilhauer et al., 2012; Houghton et al., 2013), or using modification indices to allow error terms to correlate (Houghton et al., 2013; Ciucci et al., 2014). Some authors have proposed alternative factor structures (Feilhauer et al., 2012; Houghton et al., 2013). A shorter version of this questionnaire (ICU-13) was developed in Mexico with only 13 items (Amador and Padrós, 2019). This version is shorter because there were some inconsistencies in the results found with 11 items, so these items were removed.

Morales-Vives et al. (2019) developed a new measure to assess CU traits in the Spanish population, named INventory of Callous-unemotional traits and Antisocial behavior (INCA). They reviewed the literature on CU traits in order to determine the most important facets of each trait and then wrote a pool of items for each trait, including items for all these facets. Unlike the ICU questionnaire, INCA controls the response biases social desirability (SD) and acquiescence (AC), and provides participant scores free of them. Some characteristic features of psychopathy, such as the manipulation of other people for personal gain, are considered socially undesirable, and for this reason, SD is controlled in the INCA questionnaire. Moreover, the fact of that these response biases are controlled in personality questionnaires provides a more congruent and simpler factor structure (Rammstedt and Farmer, 2013; Navarro-González et al., 2016; Morales-Vives et al., 2017). These studies have controlled these biases by applying the procedure developed by Ferrando et al. (2009) and Lorenzo-Seva and Ferrando (2009). This procedure consists of using SD item markers and content balanced items to identify a factor related to SD and AC, so that the effects of these biases can be removed from the individual scores on content factors.

The INCA questionnaire assesses the same three factors as the ICU (Callousness, Uncaring, and Unemotional). More specifically, the factor Callousness (CA) includes items about lack of empathy, guilt and remorse, and manipulation of others. The factor Unemotional (UE) includes items about lack of emotional expression. The factor Uncaring (UC) includes items about lack of responsibility and effort. The questionnaire also has an additional subscale for assessing antisocial behavior (AB) with items on challenging authority and breaking social rules. More specifically, the questionnaire has 43 Likert-type items (1 = Completely disagree, 5 = Completely agree): 4 markers of social desirability, 10 items of UE, 11 items of CA, 9 items of UC, 8 items of AB and one dummy item. More specifically, the dummy item was the first item of the questionnaire. It is a training item that can be useful for computer administrations, and it was not included in the analyses. Exploratory and confirmatory factor analysis with different samples showed the expected four-factor structure. Although several authors have correlated error terms after inspecting modification indices in order to arrive at a good fit with the ICU questionnaire, this procedure was not used with the INCA questionnaire. According to Ferrando and Lorenzo-Seva (2000), when these relaxed constraints cannot be justified for theoretical reasons, the practice becomes just an “*ad hoc*” data-driven procedure that is likely to capitalize on chance. In the case of INCA, the fit of the proposed model was considered acceptable without any further modification.

These analyses were performed using polychoric correlations because they are more appropriate for Likert items. Regarding the convergent validity of this questionnaire, the callousness, unemotional and uncaring subscales of INCA have the highest correlation with their corresponding ICU scales. Moreover, correlations were also significant with impulsivity (assessed with the BIS-11c questionnaire) and the Big Five personality traits (assessed with the OPERAS questionnaire). More specifically, UE was negatively correlated with Extraversion, CA was negatively correlated with agreeableness and positively correlated with motor impulsiveness. UC had negative correlations with conscientiousness and cognitive impulsiveness. UC and AB had positive correlations with motor impulsiveness and non-planning impulsiveness. Furthermore, AB had negative correlations with conscientiousness and agreeableness. These correlations were expected because previous studies show the relationship between CU traits and impulsivity (Roose et al., 2010; López-Romero et al., 2015; Morales-Vives et al., 2019), and also with the Big Five, especially conscientiousness and agreeableness (Miller and Lynam, 2001; Lynam et al., 2005; Essau et al., 2006; Morales-Vives et al., 2019).

It is quite common for one country to use questionnaires that have been developed in another country with the same language, sometimes without determining whether the questionnaire maintains its psychometric properties and without adjusting the vocabulary to the particular uses of each country. However, it is advisable to carry out an adaptation process that guarantees linguistic, cultural, conceptual, and metric equivalence with the original test (Muñiz et al., 2013). For this reason, and also because there are few questionnaires in Spanish that specifically assess CU traits, and even fewer that control response biases and are validated for the Mexican population, the main aim of the current study is to adapt the INCA questionnaire in Mexico (INCA-M).

As the INCA questionnaire has significant correlations with several subscales of the BIS-11c and OPERAS questionnaires, we also administered these questionnaires to see if correlations were similar with the INCA-M questionnaire. More specifically, we expected to find significant negative correlations between UE and extraversion and between CA and agreeableness. We also expected to find that both UC and AB are negatively correlated with conscientiousness. We also expected to find that UC and AB are positively correlated with the motor impulsiveness and non-planning impulsiveness of BIS-11c, and negatively correlated with cognitive impulsiveness. Finally, we expected a positive correlation between CA and motor impulsiveness. In terms of sex differences, we expected the results to be similar to those found in the study by Morales-Vives et al. (2019), in which boys obtained higher scores than girls in AG. However, it should be taken into account that the results of previous studies are contradictory: in some studies boys scored higher in CA, UC, and UE than girls (Essau et al., 2006; Fanti et al., 2009), while in others boys only scored higher on Uncaring and Unemotional (Ciucci et al., 2014). And Houghton et al. (2013) did not find significant sex differences in a sample of community children.

## Materials and Methods

### Participants

The participants were 699 adolescents aged between 12 and 18 years old (*M* = 14.8, *SD* = 1.7). There were 358 girls and 340 boys (one participant did not provide this information). Participants belonged to three different high schools in Mexico. The sampling procedure consisted of a non-random selection based on convenience. Therefore, we chose schools to which we had access. However, in order to have a heterogeneous sample, with students from different contexts and socioeconomic levels, we chose different kinds of school, located in different areas of the State of Mexico. We used probability sampling at each school, and randomly selected the classrooms. We administered the questionnaires to the students of the classrooms selected by this procedure. A total of 26.3% of students were studying the first year of lower-secondary education, 17.7% the second year of lower-secondary education, 10.6% the third year of lower-secondary education, 26.8% the first year of upper-secondary education, 10.7% the second year of upper-secondary education and 7.9% the third year of upper-secondary education.

### Measures

In addition to the INCA questionnaire, we administered several instruments to assess convergent and discriminant validity:

#### The Inventory of Callous Unemotional Traits (ICU; Frick, 2004)

This questionnaire assesses CU traits in young people: Unemotional (UE), Callousness (CA), and Uncaring (UC). We used the Spanish adaptation of this questionnaire (López-Romero et al., 2015). We carried out an exploratory factor analysis that showed the same three factors in the Mexican sample as those found in the previous Spanish study, with the following fit indices: GFI = 0.964, CFI = 0.975, RMSEA = 0.028, and RMSR = 0.0506. It has 24 items, ranging from 0 (never/almost never) to 3 (always/almost always). The internal consistencies of each subscale are α = 0.78 for UE, α = 0.76 for CA, and α = 0.82 for UC. Means and standard deviations for each subscale are: Mean = 1.4 and *SD* = 0.5 for UE, Mean = 1.2 and *SD* = 0.5 for CA, and Mean = 1.2 and *SD* = 0.6 for UC. These means and standard deviations were obtained from the mean scores and not from the sum of the items.

#### The Barratt Impulsiveness Scale – 11 for Children (BIS-11c; Chahin et al., 2010)

It has 30 Likert items ranging from 0 (never/almost never) to 3 (always/almost always). The questionnaire assesses the following subscales: motor impulsivity (MI), non-planning impulsivity (N-PI), and cognitive impulsivity (CI). We carried out an exploratory factor analysis that showed the same three factors in the Mexican sample as those found in the previous Spanish and Colombian studies, with the following fit indices: GFI = 0.957, CFI = 0.969, RMSEA = 0.028, and RMSR = 0.0523. The internal consistencies are α = 0.80 for MI, α = 0.73 for N-PI, and α = 0.68 for CI. Means and standard deviations for each subscale are: Mean = 2.2 and *SD* = 0.5 for MI, Mean = 2.4 and *SD* = 0.6 for N-PI, and Mean = 2.4 and *SD* = 0.5 for CI. These means and standard deviations were obtained from the mean scores and not from the sum of the items.

#### Overall Personality Assessment Scale (OPERAS; Vigil-Colet et al., 2013)

It contains 40 Likert items, ranging from 1 (completely Disagree) to 5 (completely agree). It assesses the Big Five personality traits: Conscientiousness (CO), Extraversion (EX), Agreeableness (AG), Emotional Stability (ES) and Openness to experience (OE), and it provides scores free of social desirability and acquiescence. More specifically, these response biases are corrected through the procedures developed by Ferrando et al. (2009) and Lorenzo-Seva and Ferrando (2009) explained above. It has good convergent validity with other personality measures and good factor reliability: ρ_*θθ*_ = 0.77 for CO, ρ_*θθ*_ = 0.86 for EX, ρ_*θθ*_ = 0.71 for AG, ρ_*θθ*_ = 0.86 for ES, ρ_*θθ*_ = 0.81 for OE. Means and standard deviations for each subscale are: Mean = 38.6 and *SD* = 11.6 for CO, Mean = 48.7 and *SD* = 9.5 for EX, Mean = 42.7 and *SD* = 11.7 for AG, Mean = 43.1 and *SD* = 11.9 for ES and *M* = 41.4 and *SD* = 10.6 for OE. These means and standard deviations are reported as T scores. We carried out an exploratory factor analysis that showed the same five factors in the Mexican sample as those found in the previous Spanish study, with the following fit indices: GFI = 0.970, CFI = 0.978, RMSEA = 0.015, and RMSR = 0.0399.

### Procedure

The Ethical Committee of the Faculty of Education Sciences and Psychology of the Universitat Rovira i Virgili approved this project. We also obtained written informed consent from all parents, in accordance with the Declaration of Helsinki. We provided information about this study to the head teachers of each school, who gave their approval.

The items were assessed by two Mexican experts in the development of personality questionnaires for adolescents. They indicated whether the statement of each item was clear and the vocabulary suitable and understandable for Mexican adolescents, taking into account the definition of each factor. On the basis of their answers, small changes were made to items 18 (“Alguna vez he tomado algo que no era mío” instead of “Alguna vez he cogido algo que no era mío”), 36 (“Es divertido hacer graffitis en las paredes” instead of “Es divertido hacer pintadas en las paredes”), and 42 (“A veces me gusta chismear sobre los demás” instead of “A veces me gusta cotillear sobre los demás”).

The questionnaires were administered collectively in groups of 25–30 students, during regular school hours, by professional psychologists. The anonymity and confidentiality of the data were guaranteed throughout the process, and participation was voluntary.

Twenty-five adolescents left the questionnaires unfinished or presented abnormal response patterns (for example, the same answers to all the items or answers in a zig zag), so they were not included in the sample. The missing data were replaced by the mean of the item. It should be taken into account that mean imputation may cause biased estimates. However, the amount of bias mostly depends on the number of missing values in the dataset which, in our case is very low (only 0.0047%). If the percentages of missing values are below 5%, the use of different imputation methods is not expected to lead to noticeable differences in the results (Lorenzo-Seva and Van Ginkel, 2016), which was the case in our study. Moreover, missing values were not concentrated on particular items. In fact, a chi-square test was performed, revealing that the missing data are equally distributed across the items, χ^2^_(__31__)_ = 37.3, *p* = 0.20.

### Data Analysis

We used the Psychological Test Toolbox (Navarro et al., 2019) to perform an Exploratory Factor analysis (EFA) and a Semi-confirmatory factor analysis. We used SPSS 25.0 for all other analyses. We did not include the first item in the analyses because it is a dummy item. We started with an EFA because a previous pilot study with 170 participants revealed that more of the antisocial behavior items loaded on the same factor than the uncaring items. For this reason, before carrying out the Semi-confirmatory factor analysis, we decided to see if the same results were obtained with the final sample. More specifically, we used Parallel Analysis to determine the number of factors to retain, and then we carried out an Exploratory Factor Analysis. After this, we carried out a Semi-confirmatory Factor Analysis using the oblique Promin rotation against a semi-specified target. We used the procedure described above to partial out the variance due to SD and AC, and unweighted least squares estimates were computed from the residual covariance.

The Psychological Test Toolbox implements the procedure for controlling response biases proposed by Ferrando et al. (2009) and Lorenzo-Seva and Ferrando (2009), which we mentioned above. The first step in this procedure consists of using the markers of SD to identify the factor defined by this bias, and the loading estimates of the markers on the SD factor are obtained by fitting the unidimensional factor analysis model to the inter-marker correlation matrix. These loading values are taken as fixed and known, and used to obtain the remaining loading values of the content items on the SD factor. Finally, once the complete SD solution has been obtained, the variance explained by the SD factor is removed from the inter-item correlation matrix. The second step consists of identifying a factor related to AC. To do so, the residual inter-item correlation matrix obtained at the end of the previous step is factor analyzed again in order to remove the variance due to acquiescent responding from the content items by using a totally or partially balanced scale (Lorenzo-Seva and Ferrando, 2009). After these two steps have been performed, the resulting residual inter-item correlation matrix is expected to reflect only item content, and is factor analyzed again to identify the content loadings of the variables of interest.

We used the Kaiser-Meyer-Olkin index (KMO; Kaiser, 1974) to find out if the data was suitable for factor analysis. We also calculated several fit indices: the Comparative Fit Index (CFI), the Goodness-of-Fit index (GFI), the Root Mean Square Residual (RMSR) and the Root Mean Square Error of Approximation (RMSEA). CFI compares the fit of a target model to the fit of an independent, or null model, while GFI tests how much better the model fits in comparison to the null model. Values of GFI and CFI higher than 0.90 indicate an acceptable fit (Bentler, 1990). RMSR indicates the average deviation of the predicted matrix from the actual correlation matrix, and values below 0.08 indicate a relatively good fit (Hu and Bentler, 1999). RMSEA is a measure of relative fit per degree of freedom, and values close to 0.06 or a stringent upper limit of 0.07 are indicative of relatively good fit (Hu and Bentler, 1999; Steiger, 2007). We also calculated the congruence indices (Tucker, 1951), which assess the similarity between the ideal loading matrix (i.e., a loading matrix in which, for each item, a single loading is one and the other loadings are zero) and the rotated loading matrix. According to Lorenzo-Seva and Ten Berge (2006), a congruence value in the range 0.85–0.94 indicates a fair similarity, while a value higher than 0.95 means that the two factors can be considered equal. Furthermore, we performed Bentler’s Simplicity index (S; Bentler, 1977) and the Loading Simplicity index (LS; Lorenzo-Seva, 2003) to evaluate the simplicity of the factor solution. The S index and the LS index range from zero to one, and a high value suggests that factor simplicity is also high. None of these indices has a cut point to help decide if factor simplicity is high. For this reason, Lorenzo-Seva (2003) suggested reporting the percentile of the value obtained in the data.

Many item scores had extreme distributions with an absolute skewness value greater than 1, and the sample sizes were also relatively large. For this reason, we decided to treat the item scores as ordered-categorical, and fit the non-linear EFA model with the inter-item polychoric correlation matrix. First, the variance due to SD and AC was partialed out using the procedure explained above. Unweighted least squares estimates were computed from the residual covariance. As the factors were expected to be correlated, the direct solution was then obliquely rotated using Promin rotation (Lorenzo-Seva, 1999). We also carried out a Semi-confirmatory Factor Analysis using the oblique Promin rotation against a semi-specified target. After that, we computed the reliabilities of the corresponding factor score estimates (see, e.g., Mellenbergh, 1994). Convergent and discriminant validity of INCA-M was assessed using the correlations between this questionnaire and other questionnaires.

Sex differences were examined with independent sample *t*-tests. In order to facilitate the interpretation of the results, the participant’s factor scores have been transformed from typical scores to T scores. Moreover, the Kolmogorov–Smirnov test indicates that the CA scores are non-normally distributed (*p* < 0.05). For this reason, in this subscale we have used the unscaled robust estimator of effect size developed by Hogarty and Kromrey (2001), instead of Cohen’s d used in the UC and UE factors (see Table 4). As Li (2016) showed, this estimator of effect size developed by Hogarty and Kromrey (2001) is generally robust to the violation of normality and homogeneity of variances.

## Results

### Item Analyses

As Table 1 shows, the means of the items ranged between 1.74 and 3.99, and standard deviations ranged between 1.13 and 2.67. Furthermore, several items had skewness and kurtosis values above 1 or below −1. This table also shows the medians, the interquartile ranges, and the percentage of participant responses on each response option.

**TABLE 1:** Means, standard deviations, medians, interquartile ranges, skewness, kurtosis, and percentage of participant responses on the five-point Likert type items of INCA-M.

							Percentage of responses				
	Mean	Standard deviation	Median	IQR	Skewness	Kurtosis	1	2	3	4	5
Item 2	3.05	1.30	3	2	–0.01	–0.97	15.7	16.5	33.6	15.9	18.3
Item 3	2.10	1.19	2	2	0.87	–0.15	42.5	23.5	21.6	6.7	5.7
Item 4	3.24	1.19	3	1	–0.21	–0.69	10.2	13.6	35.8	22.7	17.7
Item 5	3.11	2.67	3	4	–0.09	–1.58	28.2	10.1	19.0	8.4	34.3
Item 6	3.11	1.37	3	2	–0.09	–1.14	17.2	15.6	28.0	17.9	21.3
Item 7	1.74	1.15	1	1	1.54	1.43	61.9	16.9	11.9	3.7	5.6
Item 8	3.48	1.25	3	2	–0.34	–0.83	8.2	12.4	31.2	20.2	28.0
Item 9	2.01	1.69	1	2	0.97	–0.25	51.2	15.2	19.0	6.6	8.0
Item 10	3.30	1.32	3	3	–0.20	–1.00	12.2	13.6	32.3	16.0	25.9
Item 11	3.92	1.35	4	2	–1.02	–0.25	10.0	7.4	12.6	20.9	49.1
Item 12	3.68	1.14	4	2	–0.51	–0.50	4.7	9.4	29.2	26.8	29.9
Item 13	3.68	1.21	4	2	–0.58	–0.24	4.9	7.7	28.9	31.3	27.2
Item 14	2.90	1.33	3	2	0.05	–1.05	21.0	15.4	32.0	15.9	15.7
Item 15	3.99	1.31	5	2	–1.10	–0.05	8.7	7.6	11.9	19.9	51.9
Item 16	2.37	1.20	2	2	0.43	–0.66	33.5	16.7	35.6	7.6	6.6
Item 17	2.62	1.44	3	1	0.25	–0.66	23.2	19.2	38.3	10.3	9.0
Item 18	2.26	1.29	2	2	0.65	–0.74	39.9	20.9	20.0	12.2	7.0
Item 19	2.69	1.29	3	2	0.17	–0.94	26.5	13.3	36.1	12.9	11.2
Item 20	3.52	1.35	4	2	–0.52	–0.86	12.3	9.5	24.0	22.6	31.6
Item 21	2.82	1.26	3	2	0.07	–0.89	20.5	16.5	35.1	16.3	11.6
Item 22	1.84	1.64	1	2	1.31	0.39	63.0	10.6	12.6	6.6	7.2
Item 23	3.12	1.36	3	2	–0.11	–1.07	17.0	13.4	31.9	15.7	22.0
Item 24	3.19	1.43	3	2	–0.22	–1.23	19.2	12.5	23.7	20.0	24.6
Item 25	3.65	1.26	4	2	–0.55	–0.70	7.6	10.0	26.5	21.3	34.6
Item 26	3.33	1.58	3	1	–0.27	–0.81	11.1	11.4	34.3	19.7	23.5
Item 27	3.11	1.34	3	2	–0.13	–1.04	17.0	13.2	31.5	18.3	20.0
Item 28	2.97	1.34	3	2	0.05	–1.05	19.0	16.2	32.3	14.2	18.3
Item 29	3.80	1.24	4	2	–0.82	–0.23	8.2	5.4	23.3	24.6	38.5
Item 30	2.63	1.30	3	2	0.30	–0.90	26.3	18.7	31.9	11.5	11.6
Item 31	3.81	1.49	4	2	–0.80	–0.31	6.7	8.0	21.0	25.8	38.5
Item 32	3.47	1.33	4	2	–0.39	–0.94	10.9	11.6	27.9	18.6	31.0
Item 33	2.49	1.40	2	3	0.42	–1.12	36.1	16.2	22.2	14.0	11.5
Item 34	2.18	1.35	2	3	0.79	–0.61	46.6	15.5	20.3	8.2	9.4
Item 35	3.49	1.13	3	1	–0.30	–0.60	5.6	11.6	34.6	24.7	23.5
Item 36	2.15	1.82	2	2	0.86	–0.49	48.5	14.4	20.9	6.3	9.9
Item 37	3.49	1.37	4	2	–0.40	–1.06	11.5	13.0	24.7	17.0	33.8
Item 38	3.59	1.34	4	2	–0.61	–0.75	11.9	7.9	23.6	22.9	33.7
Item 39	3.00	1.27	3	2	–0.03	–0.89	16.9	14.9	35.8	16.7	15.7
Item 40	2.32	1.35	2	2	0.60	–0.85	41.5	14.3	25.0	9.2	10.0
Item 41	3.30	1.36	3	3	–0.27	–1.06	14.3	12.6	28.2	18.6	26.3
Item 42	2.62	1.37	3	3	0.33	–1.06	29.1	18.5	26.5	12.7	13.2
Item 43	2.10	1.30	2	2	0.89	–0.40	48.2	17.0	18.9	8.2	7.7

*IQR, interquartile range.*

### Factor Analysis

The KMO was 0.84, which suggests that the data is suitable for factor analysis. The first 12 consecutive eigenvalues were 7.02, 3.81, 2.84, 1.97, 1.88, 1.44, 1.28, 1.17, 1.08, 1.04, 1.00, and 0.97. Parallel analysis suggested that the data had four content factors, so we carried out an exploratory factor analysis retaining four correlated factors. The table in Supplementary Appendix 1 shows the loading values after rotation on the control scales (AC and SD) and the content scales. The results showed that all the content items except four loaded on only three factors, with the first factor corresponding to UE and the second corresponding to CA. The third included most of the items of UC and AB, although in the original study UC and AB loaded on separate factors. The GFI was 0.94 and the RMSR was 0.045. The inter-factor correlations were: 0.14 between CA and UE, 0.30 between CA and Factor 3 (UC/AB), −0.07 between CA and Factor 4, 0.10 between UE and Factor 3 (UC/AB), 0.007 between UE and Factor 4, −0.18 between Factor 3 (UC/AB) and Factor 4.

As AB items were not a factor of their own, we decided to remove these items (5, 9, 13, 17, 22, 26, 31, and 36) and fit a tri-factor solution. The solution had an appropriate fit and, once rotated, provided a structure that agreed with the expectations. We also carried out an EFA without controlling response biases, in order to determine if the procedures used to control social desirability and acquiescence might have affected the AB items. However, we obtained similar results, with most of the AB items loading on the UC factor. Therefore, controlling the response biases does not explain these results.

Taking into account these results, we carried out a Semi-confirmatory Factor Analysis using the oblique Promin rotation against a semi-specified target. The ideal pattern matrix proposed was the one obtained in the EFA, with three factors: UE, CA, and UC. Table 2 shows the loading values after rotation on the control scales (AC and SD) and the content scales, and the congruence indices for each factor. All the items loaded on the expected factor, although the loading of item 24 was lower than 0.30. However, it loaded on the expected factor, and had very small loadings on the other two factors, and a congruence index of 0.86, so we decided not to remove it. The fit indices values were CFI = 0.91, GFI = 0.91, RMSR = 0.053 and RMSEA = 0.054, so the data clearly suggest that the proposed solution is tenable. The congruence indices of the items ranged between 0.86 and 0.99. Moreover, the congruence indices of the factors ranged between 0.91 and 0.97 and the overall congruence was 0.94. Therefore, the factor similarity between the ideal loading matrix and the rotated loading matrix can be regarded as fair. Regarding the simplicity indices, the S index was 0.98 (99^th^ percentile) and the LS index was 0.51 (99^th^ percentile). These results suggest that there is a high factor simplicity, in which each item mainly loads on a single factor.

**TABLE 2:** Pattern matrix obtained in the final factor analysis, factor reliabilities, and congruence with expected factor solution.

	Control scales		Content scales			
Item	SD	AC	UE	CA	UC	Congruence
18	**0.56**	0.00	0.00	0.00	0.00	
27	**0.54**	0.00	0.00	0.00	0.00	
33	**0.51**	0.00	0.00	0.00	0.00	
42	**0.56**	0.00	0.00	0.00	0.00	
2	0.08	0.20	**0.48**	0.09	0.02	0.98
6	0.08	0.32	**0.57**	0.03	0.00	0.99
10	0.15	0.23	**−0.45**	–0.05	–0.18	0.92
14	0.14	0.25	**0.62**	0.04	0.06	0.99
19	0.09	0.25	**0.38**	0.06	0.03	0.99
23	0.22	0.38	**−0.45**	0.04	–0.11	0.97
28	0.27	0.22	**−0.51**	0.05	–0.02	0.99
32	0.11	0.47	**−0.42**	–0.05	–0.08	0.98
37	0.20	0.46	**−0.47**	–0.02	–0.11	0.97
41	0.00	0.41	**0.45**	0.10	–0.07	0.97
3	0.36	–0.13	0.08	**0.43**	0.15	0.93
7	0.22	–0.06	0.00	**0.69**	0.21	0.96
11	0.11	0.46	0.01	**−0.60**	0.05	0.99
15	–0.05	0.53	0.01	**−0.55**	0.10	0.98
20	0.09	0.43	–0.11	**−0.37**	0.03	0.96
24	0.14	0.42	–0.03	**0.20**	0.11	0.86
29	0.08	0.46	–0.16	**−0.36**	–0.04	0.90
34	0.31	–0.01	–0.04	**0.32**	0.06	0.98
38	0.09	0.40	–0.12	**−0.30**	0.03	0.92
40	0.37	0.00	–0.03	**0.30**	0.15	0.88
43	0.61	–0.14	0.03	**0.49**	0.12	0.97
4	0.31	0.14	0.09	–0.25	**0.44**	0.86
8	–0.05	0.31	0.02	–0.14	**−0.51**	0.96
12	–0.21	0.30	0.01	–0.01	**−0.31**	0.99
16	0.28	0.04	0.13	0.16	**0.43**	0.90
21	0.42	0.14	0.05	–0.21	**0.47**	0.91
25	0.28	0.35	0.03	–0.08	**−0.45**	0.98
3 30	0.16	0.03	–0.17	0.03	**0.42**	0.93
35	–0.08	0.38	–0.01	–0.05	**−0.57**	0.99
39	–0.07	0.25	0.01	0.00	**−0.42**	0.99
Reliability factor	0.78	0.78	0.80	0.77	0.72	
Congruence index			0.97	0.91	0.94	

*In bold, expected salient loadings. SD, social desirability; AC, acquiescence; UE, unemotional; CA, callousness; UC, uncaring.*

### Scale Analyses

The reliabilities of the corresponding factor score estimates are shown in Table 2. As can be seen, the values for the content scales ranged between ρ_*θθ*_ = 0.72 and ρ_*θθ*_ = 0.80. There is a relatively small number of items in the subscales, so these values can be considered to be adequate. We obtained the following correlations between the factors: 0.06 between UE and CA, 0.11 between UE and UC, and 0.12 between CA and UC.

### Convergent and Discriminant Validity

The convergent and discriminant validity of INCA-M was assessed through the correlations between this questionnaire and OPERAS, BIS-11c, and ICU. These correlations are shown in Table 3. As was expected, all the factors of INCA-M had the highest correlations with the subscales of ICU that assess the same constructs. Factor CA of INCA-M was also correlated with the factor Uncaring of ICU, and UC of INCA-M was correlated with Callousness of ICU. Moreover, UC was correlated with all the subscales of BIS-11c, especially with non-planning impulsivity. Factor CA was also correlated with motor impulsiveness, although the correlation was small. As was expected, in the OPERAS questionnaire, the factor UE was negatively correlated with the subscale Extraversion, CA was negatively correlated with Agreeableness and UC was negatively correlated with Conscientiousness. We also calculated partial correlations between the INCA-M and the other measures controlling for age and sex, without finding a relevant decrease in correlations’ size.

**TABLE 3:** Pearson correlation coefficients between INCA-M, ICU, BIS-11c, and OPERAS.

	UE	CA	UC
**ICU**			
UE	0.55**	0.10	0.19**
CA	0.10	0.32**	0.30**
UC	0.19*	0.30**	0.35**
**BIS-11c**			
MI	–0.03	0.20*	0.28**
N-PI	0.08	0.09	0.38**
CI	–0.10	0.10	−0.27**
**OPERAS**			
EX	−0.26**	0.07	–0.10
ES	−0.20*	0.01	–0.10
CO	–0.08	–0.06	−0.33**
AG	–0.12	−0.22**	–0.07
OE	–0.13	–0.17	–0.18

UE, Unemotional; CA, Callousness; UC, Uncaring; MI, Motor Impulsivity; N-PI, Non-Planning Impulsivity; CI, Cognitive Impulsivity; EX, Extraversion; ES, Emotional Stability; CO, Conscientiousness; AG, Agreeableness; OE, Openness to Experience. **p < 0.01; *p < 0.05.

**TABLE 4:** Mean scores for boys and girls on INCA-M scales and effect sizes.

	Boys	Girls	*t*	*p*	*D*
	Mean (SD)	Mean (SD)			
UE	50.6 (8.6)	49.4 (9.3)	*t*_(__696__)_ = −1.8	>0.05	–
CA	51.6 (9.7)	48.4 (8.7)	*t*_(__696__)_ = −4.8	<0.01	0.24
UC	51.5 (8.8)	48.6 (9.1)	*t*_(__696__)_ = −4.4	<0.01	0.32

*UE, Unemotional; CA, Callousness; UC, Uncaring.*

### Sex Differences

Finally, Table 4 shows the sex differences in each subscale of INCA-M (they are reported as T scores). As can be seen in this table, boys had higher scores than girls in CA and UC, and the effect size was small in CA and medium in UC.

## Discussion

The main aim of the current study was to carry out a Mexican adaptation of the INCA questionnaire and maintain its psychometric properties, because few questionnaires in Spanish assess CU traits, and even fewer are validated in the Mexican population. Moreover, the INCA questionnaire has the advantage that it controls the response biases social desirability and acquiescence, unlike other questionnaires that assess CU traits such as the ICU or the Mexican adaptation ICU-13. It should be taken into account that some characteristic features of psychopathy are considered to be socially undesirable and that the fact of controlling social desirability and acquiescence in personality questionnaires provides a more congruent and simpler factor structure (Rammstedt and Farmer, 2013; Navarro-González et al., 2016; Morales-Vives et al., 2017). For this reason, it is important to have instruments that provide participant scores that are free of these response biases.

The original INCA questionnaire, developed in Spain, assesses the same three factors as the ICU questionnaire (Unemotional, Callousness, and Uncaring), which are regarded as precursors to the development of psychopathy, and it also includes an additional factor of Antisocial Behavior with items on challenging authority and breaking social rules. However, in the Mexican adaptation only three factors have been found. The first factor contained all the items of UE and the second factor contained all the items of CA. However, most of the items referring to uncaring and antisocial behavior loaded on a common factor. This result may be explained by cultural differences, especially taking into account that the antisocial behavior items in this questionnaire are related to behaviors with little social impact (drawing graffiti on walls, being a rebel, not following the rules, etc.). In fact, the results suggest that this kind of behavior could be another sign of uncaring and irresponsible behavior in young Mexicans, while it would have more serious implications for young Spaniards. If this scale had included more extreme behaviors such as stealing, serious damage to property, etc., perhaps it would have been possible to maintain a single factor of antisocial behavior. Taking these results into account, we decided to remove these items of antisocial behavior so as to maintain the same three factors assessed by the ICU questionnaire. This prompted us to change the full name of the questionnaire in the Mexican adaptation, because the original name included the words “antisocial behavior.” In fact, the original name was INventory of Callous-unemotional traits and Antisocial behavior (INCA), and the name of the Mexican adaptation is INventory of CAllous-unemotional traits-Mexico (INCA-M).

The results indicate that INCA-M has adequate psychometric properties, with high factor simplicity and good internal consistency. Moreover, all the factors of INCA-M had the highest correlations with the subscales of ICU that assess the same constructs. The CA factor of INCA-M was also correlated with the Uncaring factor of ICU, and the UC factor of INCA-M was correlated with the Callousness factor of ICU. This result was also obtained in the original version of the INCA questionnaire (Morales-Vives et al., 2019). This result may be explained by the fact that the CA and UC subscales of ICU contain several items with very similar contents. For example, both subscales contain items related to the lack of empathy or remorse (i.e., the item “I feel bad or guilty when I do something wrong” which belongs to the UC subscale, and the item “I do not feel remorseful when I do something wrong” which belongs to CA), and both subscales contain items about the lack of responsibility (i.e., the item “I do not care about doing things well” and the item “I care about how well I do at school or work”). The correlations between INCA-M and ICU are not very high, which may be due to the unclear structure of ICU, in which similar items belong to different subscales.

The INCA-M factors also had the expected correlations with other variables linked with psychopathy, such as agreeableness and impulsivity. In fact, previous studies found a relationship between CU traits and impulsivity (Roose et al., 2010; López-Romero et al., 2015; Morales-Vives et al., 2019). This is congruent with the results of the current study, which show a lack of inhibition that is common in the aggressive and impulsive behaviors usually displayed by psychopaths. Previous studies also show that CU traits are related to the Big Five personality traits, especially conscientiousness and agreeableness (Miller and Lynam, 2001; Lynam et al., 2005; Essau et al., 2006; Morales-Vives et al., 2019). As expected, in the current study we found a negative relationship between CA and Agreeableness and a negative relationship between UC and Conscientiousness. UC involves a lack of responsibility, poor work orientation, and a lack of perseverance, and for this reason we expected to find a significant negative correlation between this factor and conscientiousness. Agreeableness refers to the tendency to be friendly and consider other’s feelings and rights, including characteristics such as empathy, cooperation, honesty, trust in others, etc. Taking into account that callousness refers to the lack of empathy and remorse, and the manipulation of others, we were expecting to find this negative correlation between them. We also found a negative correlation between UE and Extraversion, which is congruent with previous studies (Essau et al., 2006; Morales-Vives et al., 2019).

In terms of sex differences, the results found in previous studies are contradictory. Some studies found boys scored higher in CA, UC, and UE (Essau et al., 2006; Fanti et al., 2009), other studies only found they scored higher on UC and UE (Ciucci et al., 2014), Morales-Vives et al. (2019) found they scored higher only for CA, and Houghton et al. (2013) did not find significant sex differences in a sample of community children. In the current study, boys scored higher than girls on two factors: CA and UC. Clearly, then, further studies are needed to clarify the sex differences in these traits.

This study is based on a sample obtained from high schools, so further studies are needed in other samples with higher levels of antisocial behavior, such as young delinquents, to determine the predictive value of INCA-M in this kind of sample. Despite this limitation, the psychometric qualities of INCA-M are suitable for assessing UC traits in adolescents, as it shows the acceptable factor structure of the questionnaire. Moreover, the questionnaire may be useful in such fields as research, education, mental health, and forensics.

## Data Availability Statement

The datasets generated for this study are available on request to the corresponding author.

## Ethics Statement

The studies involving human participants were reviewed and approved by Ethical Committee of the Faculty of Educational Sciences and Psychology of the Universitat Rovira i Virgili. Written informed consent to participate in this study was provided by the participants’ legal guardian/next of kin.

## Author Contributions

FM-V contributed to the design of the study, carried out the statistical analyses, supervised the research, wrote most of the manuscript, and provided the final approval of the version to be published. MG-H collected the data and contacted the centers. She also wrote part of the manuscript. AV-C formulated the research question, was responsible for the statistical design of the study, supervised the research and provided the final approval of the version to be published.

## Conflict of Interest

The authors declare that the research was conducted in the absence of any commercial or financial relationships that could be construed as a potential conflict of interest.
