# Supplementary material for: INCA-M: Mexican Adaptation of the Inventory of Callous-Unemotional Traits and Antisocial Behavior
Source: Front Psychol. 2020 Apr 21;11:753. doi: 10.3389/fpsyg.2020.00753 (PMC7247810; doi:10.3389/fpsyg.2020.00753)
Supplement: Supplementary file 1 [file Table_1.DOCX]

APPENDIX 1

Pattern *matrix obtained in the exploratory factor analysis*

|  |  | Control scales | |  |  | Content scales | | |
| --- | --- | --- | --- | --- | --- | --- | --- | --- |
| Item |  | SD | AC |  | UE | CA | UC | AB |
| 18. I have occasionally taken something that is not mine  (*Alguna vez he tomado algo que no era mío*) |  | **.56** | .00 |  | .00 | .00 | .00 | .00 |
| 27. I have occasionally said bad things about other people  (*Alguna vez he dicho algo malo de alguien*) |  | **.54** | .00 |  | .00 | .00 | .00 | .00 |
| 33. I have occasionally felt jealous of somebody else  (*Alguna vez he sentido envidia de alguien*) |  | **.51** | .00 |  | .00 | .00 | .00 | .00 |
| 42. Sometimes I like to gossip about others  (*A veces me gusta chismear sobre los demás*) |  | **.56** | .00 |  | .00 | .00 | .00 | .00 |
| 2. I find it very difficult to show my feelings  (*Me cuesta mucho mostrar mis sentimientos*) |  | .08 | .22 |  | **.56** | -.07 | .08 | .09 |
| 6. I keep my feelings to myself  (*Me guardo mis sentimientos para mí mismo/a*) |  | .08 | .32 |  | **.60** | .01 | .04 | -.01 |
| 10. I like to show what I am feeling  (*Me gusta demostrar lo que siento*) |  | .15 | .20 |  | **-.44** | -.04 | -.23 | -.01 |
| 14. I hide my emotions  (*Escondo mis emociones*) |  | .14 | .26 |  | **.69** | .04 | .13 | .04 |
| 19. Other people think I am cold and distant  (*A los demás les parezco frío y distante*) |  | .09 | .25 |  | **.34** | .20 | -.07 | -.07 |
| 23. Other people can immediately tell how I am feeling  (*Los demás notan en seguida cómo me siento*) |  | .22 | .34 |  | **-.54** | .23 | .10 | .28 |
| 28. Other people say that I am very expressive  (*Los demás me dicen que soy muy expresivo*) |  | .27 | .22 |  | **-.45** | -.17 | .02 | .12 |
| 32. People can tell how I am feeling just by looking at me  (*Se me nota en la cara como me siento*) |  | .11 | .43 |  | **-.51** | -.21 | .04 | .16 |
| 37. Other people can tell at once if I am sad or angry  (*Los demás notan enseguida si estoy triste o enfadado*) |  | .20 | .43 |  | **-.58** | -.22 | -.09 | .26 |
| 41. I am reserved about my feelings  (*Soy reservado/a respecto a mis sentimientos*) |  | .00 | .37 |  | **.46** | .03 | -.07 | .08 |
| 3. I do what I like even if it might be detrimental to other  people  (*Hago lo que quiero, aunque perjudique a los demás*) |  | .36 | -.08 |  | .03 | **.30** | .19 | .29 |
| 7. I take advantage of others  (*Me aprovecho de los demás*) |  | .22 | -.01 |  | .09 | **.47** | .20 | **.41** |
| 11. I feel bad when I hurt others  (*Me siento mal cuando perjudico a otras personas*) |  | .11 | .43 |  | .08 | -.11 | -.05 | **.59** |
| 15. I feel bad when I hurt someone  (*Me siento mal cuando hago daño a alguien*) |  | -.05 | .50 |  | .07 | -.09 | -.04 | **-.57** |
| 20. Seeing other people’s misfortunes upsets me  (*Me entristece ver las desgracias de la gente*) |  | .09 | .43 |  | -.07 | **-.30** | .05 | -.24 |
| 24. I seldom apologise when I make a mistake  (*Raramente pido perdón cuando me equivoco*) |  | .14 | .38 |  | .05 | .12 | .00 | .06 |
| 29. I care about others  (*Me preocupo por los demás*) |  | .08 | .46 |  | -.10 | **-.31** | -.03 | .22 |
| 34. It is logical that clumsy people are made fun of  (*Es lógico que la gente se burle de las personas torpes*) |  | .31 | .02 |  | .09 | **.26** | .02 | .18 |
| 38. I feel bad for people who are worse off than I am  (*Me siento mal por las personas que están peor que yo*) |  | .09 | .39 |  | -.03 | **-.36** | .09 | -.15 |
| 40. I am often very pleasant with people I do not like so that I can get something out of them  (*A menudo soy muy agradable con personas que me caen*  *mal, para conseguir algo de ellas*) |  | .37 | .02 |  | -.17 | **.40** | .05 | .09 |
| 43. I sometimes use others to get what I want  (*A veces utilizo a los demás para conseguir lo que*  *quiero*) |  | .61 | -.13 |  | .07 | **.41** | .05 | .26 |
| 4. I always do things at the last minute  (*Siempre hago las cosas en el último momento*) |  | .30 | .15 |  | .00 | .08 | **.30** | **-.45** |
| 8. I try to do my work as well as I can  (*Me preocupo por hacer mis tareas de la mejor forma*  *posible*) |  | -.05 | .27 |  | .05 | -.10 | **-.54** | .06 |
| 12. I generally finish what I start  (*Generalmente acabo lo que empiezo*) |  | -.21 | .30 |  | -.10 | .22 | **-.41** | .00 |
| 16. I avoid my responsibilities  (*Evito las responsabilidades*) |  | .28 | .08 |  | .13 | .07 | **.42** | .00 |
| 21. I fall behind with my obligations  (*Me retraso en el cumplimiento de mis obligaciones*) |  | .42 | .14 |  | .03 | -.02 | **.34** | -.29 |
| 25. I like order  (*Me gusta el orden*) |  | .28 | .31 |  | -.03 | .07 | **-.50** | .04 |
| 30. I prefer to get my work done quickly, even if the result is not as good as it could be  (*Prefiero acabar mis tareas rápidamente, aunque el resultado sea peor*) |  | .16 | .03 |  | -.26 | .20 | **.30** | -.20 |
| 35. I invest time and trouble on my studies and my work  (*Invierto tiempo y esfuerzo en los estudios o en el*  *trabajo*) |  | -.08 | .36 |  | .03 | -.10 | **-.56** | .17 |
| 39. I try not to waste time  (*Evito perder el tiempo*) |  | -.07 | .25 |  | .01 | .00 | **-.42** | .13 |
| 5. I do not like the idea of taking drugs  (*Me disgusta la idea de consumir drogas*) |  | .15 | .12 |  | .08 | .02 | .04 | -.27 |
| 9. I often enjoy doing illegal things  (*A menudo me divierto haciendo cosas ilegales*) |  | .15 | -.01 |  | -.01 | .22 | **.47** | .29 |
| 13. I try to follow the rules  (*Soy una persona rebelde*) |  | -.12 | .38 |  | -.09 | .07 | **-.46** | -.21 |
| 17. I am a rebel  (*Soy una persona rebelde*) |  | .29 | .01 |  | .07 | -.18 | **.50** | .13 |
| 22. I have occasionally had legal problems  (*En alguna ocasión he tenido problemas con la ley*) |  | .28 | -.08 |  | -.09 | .24 | **.45** | .16 |
| 26. I have a great deal of respect for authority  (*Siento mucho respeto por la autoridad*) |  | -.14 | .36 |  | -.02 | .00 | **-.49** | -.05 |
| 31. I believe that the law must be respected  (*Creo que es necesario respetar las leyes*) |  | -.17 | .42 |  | -.10 | -.07 | **-.43** | .12 |
| 36. It is fun to graffiti on walls  (*Es divertido hacer graffitis en las paredes*) |  | .11 | .02 |  | .06 | .09 | **.39** | .15 |
|  |  | Eigenvalues | |  | 3.8 | 7.0 | 2.8 | 1.9 |

*Note.* SD = Social Desirability; AC = Acquiescence; UE = Unemotional; CA = Callousness; UC = Uncaring.
